# Effects of Dietary Resveratrol, Bile Acids, Allicin, Betaine, and Inositol on Recovering the Lipid Metabolism Disorder in the Liver of Rare Minnow *Gobiocypris rarus* Caused by Bisphenol A

**DOI:** 10.1155/2022/6082343

**Published:** 2022-10-07

**Authors:** Yingying Zhang, Yinan Jiang, Ziying Wang, Jiayu Wang, Mingzhen Zhu, Hui Yang

**Affiliations:** College of Animal Science and Technology, Yangzhou University, Yangzhou 225009, China

## Abstract

The fatty liver is one of the main problems in aquaculture. In addition to the nutritional factors, endocrine disrupter chemicals (EDCs) are one of the causes of fatty liver in fish. Bisphenol A (BPA) is a plasticizer widely used in the production of various plastic products and exhibits certain endocrine estrogen effects. Our previous study found that BPA could increase the accumulation of triglyceride (TG) in fish liver by disturbing the expression of lipid metabolism-related genes. How to recover the lipid metabolism disorder caused by BPA and other environmental estrogens remains to be explored. In the present study, *Gobiocypris rarus* was used as a research model, and 0.01% resveratrol, 0.05% bile acid, 0.01% allicin, 0.1% betaine, and 0.01% inositol were added to the feed of the *G. rarus* that exposed to 15 *μ*g/L BPA. At the same time, a BPA exposure group without feed additives (BPA group) and a blank group with neither BPA exposure nor feed additives (Con group) were setted. The liver morphology, hepatosomatic index (HSI), hepatic lipid deposition, TG level, and expression of lipid metabolism-related genes were analyzed after 5 weeks of feeding. The HSI in bile acid and allicin groups was significantly lower than that in Con group. The TG in resveratrol, bile acid, allicin, and inositol groups returned to Con level. Principal component analysis of TG synthesis, decomposition, and transport related genes showed that dietary bile acid and inositol supplementation had the best effect on the recovery of BPA-induced lipid metabolism disorder, followed by allicin and resveratrol. In terms of lipid metabolism-related enzyme activity, bile acid and inositol were the most effective in recovering BPA-induced lipid metabolism disorders. The addition of these additives had a restorative effect on the antioxidant capacity of *G. rarus* livers, but bile acids and inositol were relatively the most effective. The results of the present study demonstrated that under the present dosage, bile acids and inositol had the best improvement effect on the fatty liver of *G. rarus* caused by BPA. The present study will provide important reference for solving the problem of fatty liver caused by environmental estrogen in aquaculture.

## 1. Introduction

Fish fatty liver is a common disease in aquaculture and has considerable negative impact on the aquaculture industry [1]. The fatty liver could reduce the growth performance and feed utilization efficiency, reduce immunity and stress tolerance, increase susceptibility to disease and mortality [1, 2]. Recent studies have shown that the aquaculture water environment, especially the increasingly serious water pollution, is an important cause of fatty liver in aquaculture. Studies have demonstrated that organic pollutants, including environmental estrogens, insecticide, and pesticides, can induce lipid metabolism disorders in animals by interfering with their endocrine system, thereby leading to fatty liver [3, 4].

Bisphenol A (BPA) is a plasticizer that is commonly used in industry to produce materials such as polycarbonate and epoxy resins [5]. It is widely found that plastics are common in our daily lives. BPA is widely distributed in aquatic environment worldwide; the concentration of BPA could be as high as tens to hundreds of *μ*g/L, such as nearly 4 *μ*g/L in the Pearl River in China, and as high as 370 *μ*g/L in industrial wastewater in Japan [5–7]. Because of its similar structure to endogenous estrogen, BPA can combine with estrogen receptor to interfere with the endocrine system. BPA can induce oxidative stress, reduce sperm quality, inhibit egg maturation, and affect bone development of fish [8–10]. In recent years, more and more attentions have been paid to the association between BPA exposure and lipid metabolism disorders [11]. BPA exposure is closely related to human obesity, and BPA content in human urine is significantly correlated with cholesterol (TC) and triglyceride (TG) content in blood [12]. BPA can interfere with lipid metabolism, induce lipid peroxidation, and increase lipid deposition in the liver of mouse and zebrafish [13–15]. In our previous study, it was also found that BPA could induce TG level in fish liver by affecting the expression of genes related to lipid synthesis, decomposition, and transport [11]. So, the widespread distribution of BPA and its disturbance of lipid metabolism in the liver have attracted increasing attentions.

At present, the prevention and treatment of fatty liver in fish is mainly based on feed additives. The resveratrol, bile acid, allicin, betaine, and inositol are all common liver-protecting substances. Adding resveratrol to the diet of *Oncorhynchus mykiss* can increase the content of unsaturated fatty acids in the liver and reduce the content of TG [16]. The bile acids can effectively increase the expression of low-density lipoprotein mRNA, improve fat metabolism, and reduce the TG deposition in *Schizothorax prenanti* [17]. In addition, bile acids can promote the absorption of fat, improve lipid metabolism, and have a certain antioxidant effect on *Scophthalmus maximus* [18]. Allicin can significantly increase the level of high-density lipoprotein (HDL), thereby promoting lipid metabolism and reducing the levels of TG and total cholesterol in hyperlipidemia mice [19]. Betaine can improve fatty liver by promoting the synthesis of phospholipid in the liver of mouse [20]. In *Carassius auratus* and *Ctenopharyngodon idella*, the inositol can significantly reduce the lipid deposition and improve the lipid metabolism [21, 22]. However, there are few studies on whether resveratrol, bile acids, allicin, betaine, and inositol could alleviate or recover fatty liver induced by BPA.

Rare minnow *Gobiocypris rarus* is a small fish unique to China, mainly distributed in Sichuan Province and the upper reaches of the Yangtze River. Because of its small size, short generation time, and sensitivity to chemicals, it is a promising animal model for aquatic toxicological studies [23]. In this study, *G. rarus* was used as the model to explore the effects of BPA on fish hepatic lipid metabolism, and search for promising improvement measures from the perspective of feed additives. The present study will provide important reference for solving the problem of fatty liver caused by environmental pollutants in aquaculture.

## 2. Materials and Methods

### 2.1. Experimental Fish and BPA Exposure

5-month-old male *G. rarus* (weight 1.00 ± 0.30 g, body length 4.58 ± 0.39 cm) purchased from the Institute of Hydrobiology, Chinese Academy of Sciences. They were raised in glass tanks with aerated dechlorinated tap water (25 ± 1°C) and 14/10 h light/dark cycle. They were fed with chironomid larvae twice a day. There were 7 groups in total, including a blank control group (Con group), a BPA group, and 5 feed additive groups. Three repetitions were set for each group, and 21 glass tanks (20 L) were used. Twelve fish per tank, and a total of 252 fish were used in this study. 0.001% DMSO (v/v) was added into the Con group, and 15 *μ*g/L BPA (0.001% DMSO (v/v) as solvent) was added to BPA group and the 5 feed additive groups. The concentration of BPA was selected with reference to our previous studies, which was the environment-related concentration [11, 24]. Half of the water in each tank was replaced daily with fresh dechlorinated tap water dosed with the appropriate amount of BPA. The experimental duration was 5 weeks. The average BPA concentration in BPA exposed groups was 13.19 ± 1.25 *μ*g/L as determined by high-performance liquid chromatography and no BPA was detected in the Con group. Fish in Con group and BPA group were fed with basal feed. The feed additive groups were fed with feeds containing 0.01% resveratrol, 0.05% bile acid, 0.01% allicin, 0.1% betaine, and 0.01% inositol, respectively. The dosage of the additive was chosen according to the previous cyprinid fish studies [24–27]. The descriptions of the feed are shown in Table 1. No fish died during the experiment.

### 2.2. Sampling and Determination of Hepatosomatic Index (HSI)

Before dissection, the body weight and body length of *G. rarus* were measured. Following dissection, the liver tissue was weighed and the HSI was calculated. For each treatment group, the liver from six fish was taken for mRNA expression analysis (two fish per replicate in triplicate), the liver from six fish was used for oil red O staining (two fish per replicate in triplicate), the liver from six fish was used for triglyceride (TG) analysis (two fish per replicate in triplicate), and the liver from eighteen fish was used for enzyme activities analysis (six fish per replicate in triplicate). After quick freezing with liquid nitrogen, they were stored at -80°C until use.

### 2.3. Oil Red O Staining

The tissues were washed with phosphate buffer solution (PBS), soaked in 30% sucrose solution at 4°C overnight, and then sliced at -20°C with a frozen microtome. The slices were washed slightly in 60% isopropanol and then stained with oil red O for 15 min. The stained sections were washed with 60% isopropanol to remove the excess dye solution, washed under tap water, and then stained with hematoxylin for 2 min. Wash the slices under tap water until the nuclei were blue, wiped off the excess water, sealed them with glycerin gelatin, and take photos under the microscope. Microscopic examination was carried out using CHC binocular microscope (Olympus).

### 2.4. Determination of TG Content

Liver tissues were homogenized using PBS and then centrifuged at 1654 g for 10 min to separate the supernatant. TG determination was carried out with a commercial kit (Nanjing Jiancheng). The standards and liver supernatant samples were added into the 96-well microplate with prefabricated reagent, respectively. After incubation at 37°C for 10 min, microscopic examination was carried out using fluorescence microscope (Olympus) with the excitation light wavelength at 510 nm. The protein concentrations were assayed using the commercial protein assay kit (Beyotime). Concentration of final level TG was normalized to protein concentration of the corresponding sample.

### 2.5. Real-Time PCR Detection of Lipid Metabolism-Related Genes

Total RNA of liver tissue was isolated by trizol one-step method according to the instruction book (CoWin). RNA integrity was checked by analyzing 28S and 18S rRNA ratios. The concentration and purity of the isolated RNA were measured by spectrophotometer (Thermo). The cDNAs were synthesized from total RNA using oligo (dT)18 primer and random primer with M-MLV reverse transcriptase and gDNA wiper (Vazyme, China). qPCR was applied to evaluate mRNA expression profiles of lipid metabolism-related genes. These genes were including TG lipidolysis related genes: phosphoglycerate kinase 1 (*pgk1*), glycerol-3-phosphate dehydrogenase 1a (*gpd1a*), and triosephosphate isomerase (*tpi*); TG synthesis related genes: glycerol-3-phosphate acyltransferase (*gpat3* and *gpat4*) and diacylglycerol acyltransferase (*dgat1a* and *dgat1b*); and TG transport related apolipoproteins (*apoB100*, *apoCI*, *apoCII*, *apoE*). Actin beta (*actb*) and eukaryotic translation elongation factor 1 alpha (*ef1a*) were selected as the reference genes. The primers are listed in Table S1. The qPCR efficiency (*E*) of each PCR reaction was calculated, and the *E* values were all between 90% and 110%. The relative expression level was calculated using 2^-△△Cq^ method.

### 2.6. Lipid Metabolism Enzyme and Antioxidant Enzyme Activities Analysis

Liver samples were homogenized with PBS. The grinding fluid was transferred and divided into several aliquots for subsequent measurement. The activities of lipid metabolism enzymes including acetyl-CoA carboxylase (ACC), fatty acid synthase (FASN), glycerol-3-phosphate acyltransferase (GPAT), and carnitine palmitoyltransferase 1 (CPT1), and the activities of antioxidant enzymes including catalase (CAT), superoxide dismutase (SOD), and glutathione peroxidase (GPX), as well as the total antioxidant capacity (T-AOC), were determined with commercial kits (Nanjing Jiancheng) following the manufacturer's protocol. Concentrations of final activities of enzymes were normalized to protein concentrations of the corresponding samples. The total protein concentrations of the samples were determined with protein quantitation kit (Beyotime).

### 2.7. Data Analysis

All statistical data were expressed as mean ± SD. Data were tested for normality of distribution (Shapiro-Wilk test) prior to analysis. Data that did not meet normality were transformed (lg) and then analyzed by Student's *t*-test (between Con and the treatment groups). *P* < 0.05 was regarded as significant differences between treated and Con groups. The statistical analyses were performed with the SPASS18.0. Data analysis was performed using SPASS 18.0. Principal component analysis (PCA) of the gene expression was performed by Origin 2021.

## 3. Results

### 3.1. Liver Morphology, Body Weight, Liver Weight, and HSI of *G. rarus*

**Figure 1:**
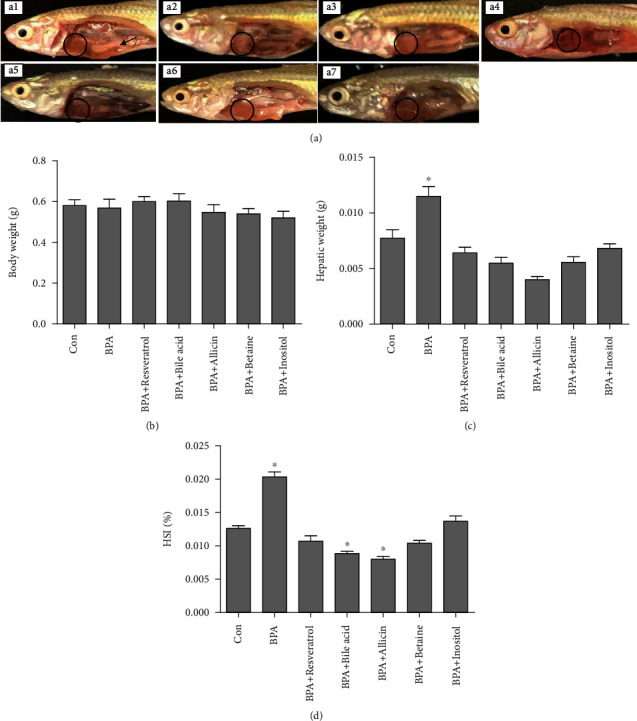
Effect of the experimental additives on rare minnow hepatic morphology, body weight, hepatic weight, and HSI. (a) Hepatic morphology, a1: Con, a2: BPA, a3: resveratrol, a4: bile acid, a5: allicin, a6: betaine; a7: inositol; (b) body weight; (c) hepatic weight; (d) HSI. The circle shows part of the liver, and the arrow points to the testis. All statistical data were expressed as mean ± SD (*n* =3).∗ stands for there is a significant difference between the treatment groups and Con groups (*P* < 0.05).

Hepatic morphology showed that the liver of BPA and betaine groups was significantly whiter than that of the Con group. The liver of resveratrol group was slightly whiter than that of the Con group, while the liver of bile acid, allicin, and inositol groups was ruddy (Figure 1(a)). As shown in Figure 1(b), there was no significant change in body weight of *G. rarus*. Compared with the Con group, the liver weight of BPA group increased significantly, while the liver weight of resveratrol, bile acid, allicin, betaine, and inositol groups all returned to the Con level (Figure 1(c)). HSI of BPA group was significantly higher than that of Con group, while the HSI of resveratrol, betaine, and inositol groups returned to the Con level. The HSI of bile acid and allicin groups was significantly lower than that of Con group (Figure 1(d)).

### 3.2. Hepatic Lipid Accumulation

**Figure 2:**
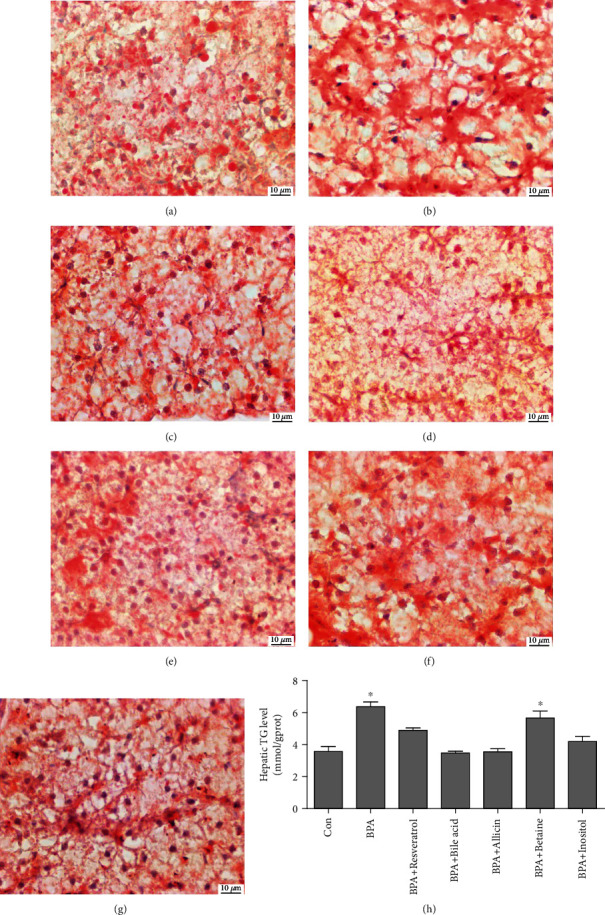
Hepatic oil red O staining and TG levels. (a) Liver from Con group; (b) liver from BPA treatment group; (c) liver from resveratrol group; (d) liver from bile acid group; (e) liver from allicin group; (f) liver from betaine group; (g) liver from inositol group; (h) effect of the experimental additives on rare minnow hepatic TG level. The red part represents lipids. All statistical data were expressed as mean ± SD (*n* =3). ∗ stands for there is a significant difference between the treatment groups and Con groups (*P* < 0.05).

Compared with the Con group, the oil red O staining of the liver in the BPA group was deepened, indicating that BPA significantly increased the liver lipid deposition of *G. rarus*. Under BPA exposure, the liver lipid deposition of *G. rarus* fed with resveratrol, bile acid, allicin, and inositol decreased compared with the BPA group, indicating that the additions can significantly mitigate the liver lipid deposition of *G. rarus* caused by BPA. The liver lipid deposition in betaine group was still increased compared with the Con group, indicating that betaine at the present dosage could not mitigate the hepatic lipid deposition caused by BPA (Figures 2(a)–2(g)). Consistent with the results of oil red O staining, the TG content of BPA and betaine groups increased significantly compared with the Con group (increased by 1.78 times and 1.58 times, respectively), while the TG level of resveratrol, bile acid, allicin, and inositol groups had no significant difference compared with the Con group (Figure 2(h)). These results suggest that resveratrol, bile acid, allicin, and inositol at the present dosage can significantly mitigate the lipid deposition in the liver of *G. rarus* caused by BPA.

### 3.3. Expression of Lipid Metabolism-Related Genes

**Figure 3:**
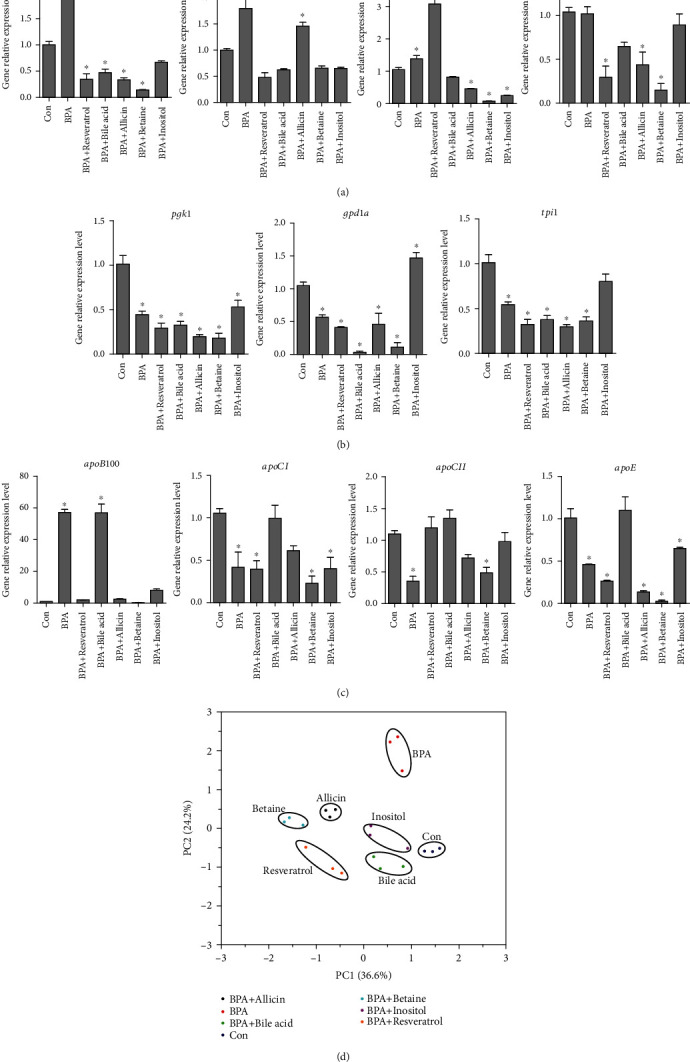
mRNA expression of lipid metabolism-related genes of rare minnow following BPA and experimental additives treatment. (a) TG lipidolysis related genes; (b) TG synthesis related genes; (c) TG transport related genes; (d) the PCA analysis results of lipid metabolism-related genes. All statistical data were expressed as mean ± SD (*n* =3). ∗ stands for there is a significant difference between the treatment groups and Con groups (*P* < 0.05).

**Figure 4:**
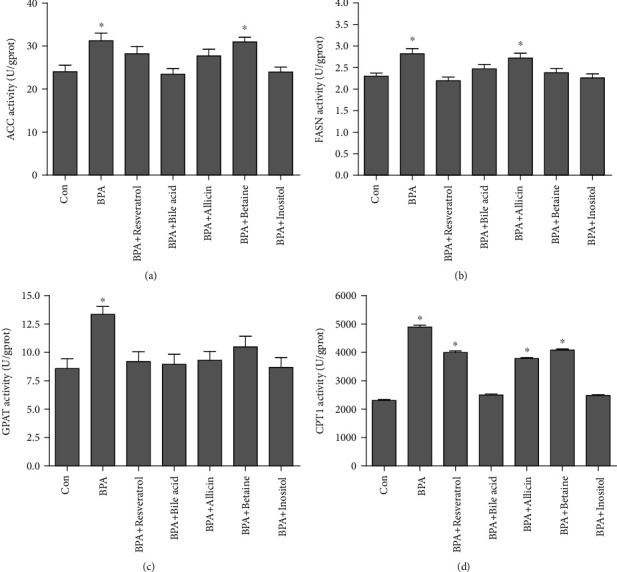
Lipid metabolism enzyme activities in rare minnow liver following BPA and experimental additives treatment. (a) ACC; (b) FASN; (c) GPAT; (d) CPT1. All statistical data were expressed as mean ± SD (*n* =3). ∗ stands for there is a significant difference between the treatment groups and Con groups (*P* < 0.05).

The *pgk1*, *gpdla*, and *tpil* genes were all involved in the decomposition of TG. As shown in Figure 3, the expression of *pgk1* in all BPA containing groups decreased significantly (1.9-5.56 times) compared with the Con group, but the decrease in inositol group was less than that in other treatment groups. The expression level of *gpdla* in inositol group was significantly higher than that in Con group (increased by 1.4 times), while decreased significantly in resveratrol, bile acid, allicin, and betaine groups compared to Con group (decreased by 1.85-9.33 times). The expression of *tpil* in inositol group did not change significantly compared with Con group, but decreased significantly in BPA, resveratrol, bile acid, allicin, and betaine groups (decreased by 1.85-3.4 times) (Figures 3 and 4). The expression of TG decomposition related genes suggests that inositol at the present dosage added to feed had a better effect on the recovery of BPA-induced lipid metabolism disorder.

The *gpat3*, *gpat4*, *dgat1a*, and *dgat1b* genes participated in the synthesis of TG. The expression of *gpat3* increased significantly in BPA group (increased by 1.98 times) but decreased significantly in resveratrol, bile acid, allicin, and betaine groups (2.12-7.12 times). The expression of *gpat3* in inositol group had no significant change compared with the Con group. *gpat4* expression increased significantly in BPA and allicin groups (1.8 and 1.45 times, respectively) and returned to the Con level in resveratrol, bile acid, betaine, and inositol groups. The expression of *dgat1a* increased significantly in resveratrol group (2.93 times) but decreased significantly in allicin, betaine, and inositol groups (2.3-13.82 times). Compared with the Con group, the expression of *dgat1b* in resveratrol, allicin, and betaine groups decreased significantly (by 2.37-7 times) (Figure 3(b)). Expression of TG synthesis related genes suggests that the addition of bile acid and inositol at the present dosage in the feed had a better effect on the recovery of lipid metabolism disorder caused by BPA.

**Figure 5:**
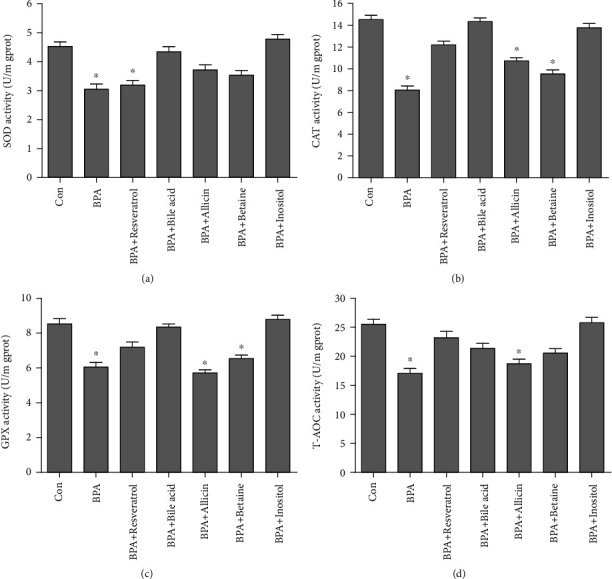
Antioxidant enzyme activities in rare minnow liver following BPA and experimental additives treatment. (a) SOD; (b) CAT; (c) GPX; (d) T-AOC. All statistical data were expressed as mean ± SD (*n* =3). ∗ stands for there is a significant difference between the treatment groups and Con groups (*P* < 0.05).

The expression of *apoB100* increased significantly in BPA and bile acid group (53.34 and 53.06 times, respectively), while resveratrol, allicin, betaine, and inositol groups all recovered to the Con level (Figure 5(a)). The expression of *apoCI* in BPA, resveratrol, betaine, and inositol groups was significantly lower than that in Con group (2.53-4.62 times), while bile acid and allicin returned to Con level (shown in Figure 5(b)). The expression of *apoCII* decreased significantly in BPA and betaine group (3.11 times and 2.26 times, respectively) and returned to Con level in resveratrol, bile acid, allicin, and inositol groups (Figure 5(c)). Compared with the Con group, the expression of *apoE* in bile acid group did not change significantly but decreased significantly (1.56-38.88 times) in the BPA, resveratrol, allicin, betaine, and inositol groups (Figure 5(d)). Expression of TG transport related genes suggests that the addition of bile acid, allicin, and inositol at the present dosage had a better effect on the recovery of lipid metabolism disorder caused by BPA.

The PCA of lipid metabolism gene expressions showed that gene expression of BPA group was significantly different from that of the other groups. The gene expression changes in bile acid and inositol groups were most similar to those in the Con group. The gene expression changes in allicin, resveratrol, and betaine groups were the most different from those in the Con group. This indicated that the addition of bile acid and inositol at the present dosage in the feed had the best effect on the recovery of lipid metabolism disorder caused by BPA, while betaine at the addition dosage in the present study had the worst effect on the recovery of lipid metabolism disorder caused by BPA.

### 3.4. Activity of Lipid Metabolism-Related Enzymes

ACC, FASN, and GPAT activities in *G. rarus* liver tissues were significantly increased under BPA exposure (Figure 4) (*P* < 0.05). ACC activity returned to Con level in the resveratrol, bile acid, allicin, and inositol groups, but remained significantly higher in the betaine group than in the Con group (Figure 4(a)) (*P* < 0.05). FASN activity returned to Con level in all treatment groups except the allicin group (Figure 4(b)), and GPAT activity returned to Con level in all treatment groups (Figure 4(c)). CPT1 activity returned to Con level in the bile acid and inositol groups, while it remained significantly higher in the resveratrol, allicin, and betaine groups than in the Con group (Figure 4(d)) (*P* < 0.05). In terms of lipid metabolism-related enzyme activity, bile acids and inositol at the present dosage were the most effective in alleviating BPA-induced lipid metabolism disorders, followed by resveratrol.

### 3.5. Activity of Antioxidant Enzymes

SOD, CAT, and GPX activity in *G. rarus* liver tissues were significantly reduced under BPA exposure alone, and T-AOC was also significantly inhibited (Figure 5) (*P* < 0.05). SOD activity returned to Con levels in the bile acid, allicin, betaine, and inositol groups, while it remained significantly reduced in the resveratrol group (Figure 5(a)) (*P* < 0.05). CAT activity returned to Con levels in the resveratrol, bile acid, and inositol groups, while it remained significantly lower in the allicin and betaine groups (Figure 5(b)) (*P* < 0.05). The change in GPX activity was consistent with that of CAT (Figure 5(c)). T-AOC remained significantly reduced in the allicin group (*P* < 0.05), while it returned to Con levels in all the remaining groups (Figure 5(d)). Overall, the addition of these species had a restorative effect on the antioxidant capacity of *G. rarus* liver, but at the present dosage, bile acids and inositol were the most effective, followed by resveratrol.

## 4. Discussion

Our previous studies have demonstrated that BPA exposure can disrupt *G. rarus* hepatic lipid metabolism, resulting in increased TG accumulation [11]. How to alleviate the negative effects of hepatic lipid metabolism caused by BPA is very meaningful. In the present study, based on the comprehensive result of TG level, oil red O staining, and expression of genes related to lipid metabolism, inositol exhibited the most effective for alleviating liver lipid accumulation induced by BPA exposure, followed by bile acid, resveratrol, and allicin. However, betaine had no significant alleviation effects on the accumulation of TG in the liver at the dosage in the present experiment. Inositol is a vitamin substance and widely distributed in animals and plants [28]. Many studies have revealed the role of inositol in reducing lipid deposition in tissues [29, 30]. Inositol deficiency could be associated with a variety of lipid metabolic disturbances resulting in accumulation of lipids in the liver and decreased hepatic lipoprotein output [31, 32]. The dietary supplementation with inositol could reduce hepatic TG accumulation in rats [33]. So, it will not be doubtful that inositol can reduce the content of TG in the liver in the present study.

The relative expression level of lipid metabolism-related genes including TG decomposition, synthesis, and transport genes was also detected under different treatments. The expression profiles of these genes were different, but the PCA analysis indicated the bile acid and inositol groups were most similar to those in Con group. The expression changes of lipid metabolism-related genes induced by BPA exposure are well understood [34–36]. The addition of bile acids and inositol could counteract the increase in TG content caused by BPA and ultimately maintain the similar TG content as the Con group, which may be closely related to regulating the expression of these genes.

ACC, FASN, and GPAT enzymes are mainly involved in the synthesis of fatty acids [37, 38]. Previous studies have demonstrated that the elevation of these enzymes could significantly increase the deposition of TG in the body and lead to obesity [39, 40]. In the present study, the activities of these enzymes were significantly increased under BPA exposure, which could promote the accumulation of hepatic lipids and lead to the production of fatty liver. The up-regulation of these enzymes by BPA had also been found in other studies [41, 42]. The addition of bile acid and inositol had the best alleviation effects on lipid metabolism disorders caused by BPA. The inhibition of these enzymatic activities would help to alleviate the accumulation of TG in the liver caused by BPA. But the specific regulatory mechanisms of bile acid and inositol on these enzymes had not been reported yet.

It has been reported that BPA could increase the level of ROS and cause oxidative stress in fish [43, 44]. Oxidative stress brings a variety of hazards, and it is necessary to remove excess ROS by increasing the activity of antioxidant enzymes in the body [45]. In the present study, BPA exposure could significantly inhibit the activities of these antioxidant enzymes including SOD, CAT, GPX, and T-AOC, suggesting that ROS balance was disrupted, which would cause oxidative stress in *G. rarus.* However, after the addition of bile acid and inositol, the activities of these antioxidant enzymes did not change significantly compared with the Con, indicating that they can improve the ROS imbalance induced by BPA exposure. At present, there are also reports about the ability of bile acid and inositol to improve the body's antioxidant capacity. The inositol could enhance antioxidant status and depress oxidative damage in *Cyprinus carpio* enterocytes [46]. The pretreated with diverse levels of inositol could influence the survival, immune response, and antioxidant status of *Litopenaeus vannamei* and *C. carpio* [47, 48]. The dietary supplementation of bile acid could attenuate adverse effects of high-fat diet on growth performance, antioxidant ability, lipid accumulation, and intestinal health in *Micropterus salmoides* [49]. But it does not mean that other additives have no effects on these antioxidant enzymes. In fact, resveratrol and allicin also exhibited certain effects. The effect of betaine on these antioxidant enzymes was similar to that of BPA treatment, which may be related to the dosage of betaine added in the present study.

In conclusion, the present study examined the effect of supplementation of resveratrol, bile acid, allicin, betaine, and inositol on BPA-induced fatty liver in *G. rarus.* When fed with resveratrol, bile acid, and inositol, the content of TG in the liver could return to Con levels. Combined with the results of the relative expression of lipid metabolism-related genes, lipase activity, and antioxidant enzyme activity, the inositol and bile acid have the best effects on relieving lipid accumulation in the liver, at the present dosage, the effects of resveratrol and allicin are general, while betaine had no obvious improvement effects. The results of the present study will provide an important reference for further solving the lipid accumulation in fish liver in aquaculture.

## Figures and Tables

**Table 1:** Formulation and composition of experimental diets (%).

Feed ingredients	The proportion of each ingredient (%)					
	Con group	Resveratrol group	Bile acid group	Allicin group	Glycine betaine group	Inositol group
Fish meal	10	10	10	10	10	10
Soybean meal	18	18	18	18	18	18
Cottonseed meal	18	18	18	18	18	18
Tapioca flour	5	5	5	5	5	5
Soybean oil	4	4	4	4	4	4
Premix ^a^	4.1	4.1	4.1	4.1	4.1	4.1
Lecithin oil	1	1	1	1	1	1
Methionine	0.1	0.1	0.1	0.1	0.1	0.1
Flour	39.8	39.79	39.75	39.79	39.7	39.79
∗Experimental additive	0	0.01	0.05	0.01	0.1	0.01
Total	100	100	100	100	100	100

^a^Premix: vitamin and mineral, respectively (mg/kg): VA: 28 mg, VB1: 12 mg, VB2: 12 mg, VB6: 14.4 mg, VB12: 0.2 mg, VE: 300 mg, VK3: 20 mg, VD: 10 mg, VC: 600 mg, ascorbic acid: 1000 mg, VE: 400 mg, nicotinamide: 80 mg, calcium pantothenate: 100 mg, biotin: 0.4 mg, CuSO_4_·5H_2_O: 10 mg, FeSO_4_·H_2_O: 300 mg, ZnSO_4_·H_2_O: 300 mg, MnSO_4_·H_2_O: 100 mg, Na_2_SeO_3_: 10 mg, CoCl_2_·6H_2_O (10% Co): 2 mg, NaCl: 100 mg, zeolite: 200 mg, MgSO_4_: 500 mg.

## Data Availability

The data that support the findings of this study are available from the corresponding author upon reasonable request.

## Supplementary Materials

Supplementary Materials Table S1 Primer sequences of qRT-PCR.
