# Supplementary material for: Effects of Dietary Resveratrol, Bile Acids, Allicin, Betaine, and Inositol on Recovering the Lipid Metabolism Disorder in the Liver of Rare Minnow Gobiocypris rarus Caused by Bisphenol A
Source: Aquac Nutr. 2022 Oct 7;2022:6082343. doi: 10.1155/2022/6082343 (PMC9973200; doi:10.1155/2022/6082343)
Supplement: Supplementary Materials — Table S1 Primer sequences of qRT-PCR. [file 6082343.f1.doc]

**Supplemental Materials**

Table S1 Primer sequences of qRT-PCR.

| **Gene** | **Name** | **Sequence** | **Genbank No.** |
| --- | --- | --- | --- |
| *actb* | F | GTCCGTGACATCAAAGAG | OP185532 |
|  | R | ACCGCAAGATTCCATAC |  |
| *apoB100* | *F* | TTGGAACAGTGGGAAGTGGA | OP185530 |
|  | R | ATCAACAGTAATGTGGCTCAGG |  |
| *apoCI* | F | CAAAAGTTGGATCAGAGCGAGT | JX649103.1 |
|  | R | TGGAGGGTCACATATCAGGAAG |  |
| *apoCII* | F | CACTGCCAGCGGCTATGTA | OP185531 |
|  | R | CCGTCTTCTTGCGGGATG |  |
| *apoE* | F | ACACCGTGGCAACCTATCTG | MZ606378 |
|  | R | TCCTCTGCGGTCTGTTCCA |  |
| *dgat1a* | F | TCGCCGTCTTCTTCCTGTC | OP185525 |
|  | R | GCTGCGTTCCCATAGTTTCC |  |
| *dgat1b* | F | GGGATTTCTGTTCAGGAGGC | OP185526 |
|  | R | AGAGGAGATGGTTGGGCACT |  |
| *ef1a* | F | ACAAATGCGGTGGAATCG | OP185533 |
|  | R | TCAAACTTCCAGAGAGCGATA |  |
| *gpat3* | F | GGCTATCCGTAGTAGTGGGG | OP185523 |
|  | R | GTGTGTCCGTCGCAGTTCTC |  |
| *gpat4* | F | GCTATGGTGGGTCAGGTCC | OP185524 |
|  | R | TGCAACATGGTCACTTAATCG |  |
| *gpd1a* | F | GCTAAGATAGTTGGCACGAATG | OP185528 |
|  | R | GTCACAAGCACGACCGATG |  |
| *pgk1* | F | AGTGTTGCCTGGTGTTGATG | OP185527 |
|  | R | AGCGGAGTAGTTGTCTTTTGC |  |
| *tpi1* | F | GTGCTCCACCCATCTACCTC | OP185529 |
|  | R | CACACCCAAACCATTCTCAAG |  |
